# Neuroprotective effects of hyperbaric oxygen (HBO) therapy on neuronal death induced by sciatic nerve transection in rat

**DOI:** 10.1186/s12883-017-1004-1

**Published:** 2017-12-16

**Authors:** Zahra Shams, Ali Reza khalatbary, Hassan Ahmadvand, Zohreh Zare, Kosar Kian

**Affiliations:** 1 Molecular and Cell Biology Research Center, Department of Anatomy, Faculty of Medicine, Mazandaran University of Medical Sciences, Sari, Iran; 2 Department of Biochemistry, Faculty of Medicine, Lorestan University of Medical Sciences, Khorramabad, Iran; 3 Razi Herbal Researches Center, Lorestan University of Medical Sciences, Khorramabad, Iran

**Keywords:** Hyperbaric oxygen, Nerve transaction, Apoptosis, Inflammation

## Abstract

**Background:**

Recent studies shows that hyperbaric oxygen (HBO) therapy exerts some protective effects against neural injuries. The purpose of this study was to determine the neuroprotective effects of HBO following sciatic nerve transection (SNT).

**Methods:**

Rats were randomly divided into five groups (*n* = 14 per group): Sham-operated (SH) group, SH + HBO group, SNT group, and SNT + pre- and SNT + post-HBO groups (100% oxygen at 2.0 atm absolute, 60 min/day for five consecutive days beginning on 1 day before and immediately after nerve transaction, respectively). Spinal cord segments of the sciatic nerve and related dorsal root ganglions (DRGs) were removed 4 weeks after nerve transection for biochemical assessment of malodialdehyde (MDA) levels in spinal cord, biochemical assessment of superoxide dismutase (SOD) and catalse (CAT) activities in spinal cord, immunohistochemistry of caspase-3, cyclooxigenase-2 (COX-2), S100beta (S100ß), and terminal deoxynucleotidyl transferase dUTP nick end labeling (TUNEL) in spinal cord and DRG.

**Results:**

The results revealed that MDA levels were significantly decreased in the SNT + pre-HBO group, while SOD and CAT activities were significantly increased in SNT + pre- and SNT + post-HBO treated rats. Attenuated caspase-3 and COX-2 expression, and TUNEL reaction could be significantly detected in the HBO-treated rats after nerve transection. Also, HBO significantly increased S100ß expression.

**Conclusions:**

Based on these results, we can conclude that pre- and post-HBO therapy had neuroprotective effects against sciatic nerve transection-induced degeneration.

## Background

After peripheral nerve transection, a proportion of sensory and motor neurons progressively die through apoptosis [1–3]. Therefore, it has been thought that the survival of the axotomized populations of neurons is the prime issue to restore the function of target organs [4]. Several mechanisms account for the apoptosis of neuronal cells after nerve transection, including excitotoxicity, alternations in electrical activity, oxidative stress, neurotrophic support deficit, neurotoxic inflammatory products, and alternation in cellular homeostasis [5–7]. Therefore, it has been postulated that the use of free radical scavengers [8], anti-inflammatory agents [9], and nerve growth factors [10] may offer some protection against neural apoptosis after peripheral nerve transection. Due to the complexity of the nerve cell destructive processes after nerve transection, a satisfactory therapeutic method has not been developed yet. So, finding an appropriate therapeutic method to reduce this process is an important issue in field of peripheral nerve regeneration.

Hyperbaric oxygen (HBO) therapy, treatment providing 100% oxygen at a pressure greater than that at sea level, can be considered as one of these methods. In this regard, studies documented that HBO have neuroprotective effects against traumatic brain and spinal cord injury [11, 12], ischemic brain and spinal cord injury [13, 14], neurodegenerative disorders [15, 16], oxidative damage in neuronal culture [17], and neuropathic pain [18]. Recently, HBO has been demonstrated to improve nerve regeneration [19, 20] following peripheral nerve injury. The beneficial effects can be attributed to some biological activities such as anti-oxidative [21, 22], anti-inflammatory [23, 24], and anti-apoptotic [25, 26] properties. Also, it was documented that HBO increased oxygen supply [27] and improved neural metabolism [28] after ischemia, along with promoting thrombolysis [29]. Despite the neuroprotective effects of HBO therapy against various experimental models of neural injury and disease, no studies have been conducted on the influence of HBO on neural apoptosis after nerve transection. Accordingly, we investigated the beneficial effects of HBO therapy on neuronal cell preservation after sciatic nerve transection.

## Methods

### Animals

A total of 70 adult male Sprague-Dawley rats were used in this study (laboratory animal research center, Sari, Iran). The animals were kept in the laboratory under constant conditions of light/dark cycle (12 h/12 h) and temperature (23 ± 1 °C). Experimental procedures and protocols used in this study were approved by ethical committee of Health Sciences, Mazandaran University of Medical Sciences (IR.MAZUMS.REC.1396.2978).

### Nerve transection and experimental design

Under anesthesia with intraperitoneal ketamine (60 mg/kg) plus xylazine (10 mg/kg), sciatic nerve was transected unilaterally (right side) at the level of the sacrotuberous ligament. Spontaneous regeneration of sciatic nerve was inhibited by excising a 3 mm segment of distal nerve stump, and reflection of the distal and proximal ends of the transected nerves [30]. The rats were placed in HBO chamber, the pressure was gradually raised to and maintained at 2.0 atm absolute (at a rate of 0.1 ATA/min), then allowed to breathe 100% oxygen for 60 min per day and decompressed to normal room pressure at a rate of 0.1ATA/min [31, 32]. All rats in the same group were kept in the chamber for 30 min to adapt it to the experimental conditions and then underwent treatment at the same time.

The animals were randomly allocated in five groups, each containing 14 rats: (Ι) Sham-operated (SH) group (underwent skin suture alone); (ΙΙ) SH + HBO group (underwent skin suture and received HBO); (III) sciatic nerve transection (SNT) group (underwent skin suture followed by sciatic nerve transection); (IV) SNT + pre-HBO group (HBO treatment beginning on 1 day before nerve transection and followed for 5 days); (V) SNT + Post-HBO group (HBO treatment for 5 days beginning on immediately after nerve transection and recovery from anesthesia).

Four weeks after nerve transection, each group of animals was divided into 2 subgroups: (A) in which rats (*n* = 7) were euthanized with an intraperitoneal injection of overdose of sodium pentobarbital, and spinal cord segments of the sciatic nerve removed from vertebral column for biochemical analysis, (B) in which rats (*n* = 7) were euthanized with an intraperitoneal injection of overdose of sodium pentobarbital, and spinal cord segments of the sciatic nerve and related dorsal root ganglions (ipsilateral L4 and L5 DRGs) removed for histopathological assessment and immunohistochemistry. The doses and treatment schedules were based on previous studies [29–31] and pilot experiments in our laboratory.

### Biochemistry

Four weeks after nerve transection, the obtained spinal cord samples were immediately frozen and stored in a − 80 °C freezer for assays of tissue malondialdehyde (MDA) levels as a product of lipid peroxidation, and catalase (CAT) and superoxide dismutase (SOD) activities. Thiobarbituric acid reactive substances measurement was used to calculate of MDA level as micromoles per milligram of protein [33]. Catalase (CAT) enzyme activity was measured spectrophotometrically based on the reaction of the enzyme with methanol in the presence of hydrogen peroxide and expressed as unit per milligram of protein [34]. The estimation of superoxide dismutase (SOD) activity was based on the inhibition of superoxide radical reaction with pyrogallol which was determined spectrophotometrically by the absorbance at 420 nm and expressed as unit per milligram of protein [35].

### Histopathology

Four weeks after nerve transection, the obtained spinal cord segments and related dorsal root ganglions were immediately fixed in 10% neutral buffered formalin. Five-micrometer serial transverse sections were taken from the paraffin-embedded blocks, deparaffinized, and then stained with Cresyl violet to assess the histopathological changes. All the histological assessments were done in a blinded fashion.

### Immunohistochemistry

For immunohistochemistry, some sections were incubated with anti-caspase 3 rabbit polyclonal antibody (1:200 in PBS, *v*/v, Abcam), anti-COX 2 rabbit polyclonal antibody (1:200 in PBS, v/v, Abcam), and anti-S100ß rabbit polyclonal antibody (1:500 in PBS, v/v, Abcam) overnight at 4 °C. Sections were washed with PBS and incubated with secondary antibody conjugated with horseradish peroxidase (goat anti-rabbit IgG, Abcam) for 2 h. For quantitative analysis, immunohistochemical photographs (n = five photos from each five-micrometer serial transverse sections of ipsilateral spinal cord segments of the sciatic nerve and related dorsal root ganglions, the thickness of between sampled sections was 48 μm for spinal cord and 36 μm for DRG) from all rats in each experimental group were assessed by densitometry using ImageJ software. Data are expressed as a percentage of total tissue area.

### TUNEL staining

TUNEL staining on some sections was performed using a TUNEL detection kit (Roche). Briefly, the sections incubated for 10 min with 3% H2O2 and for 15 min with proteinase-K, and then incubated with TUNEL reaction mixture for 60 min at 37 °C. In the following, the samples were incubated for 30 min with converter POD at 37 °C and demonstrated with DAB for 10 min. For quantitative analysis, immunohistochemical photographs (n = five photos from each five-micrometer serial transverse sections of ipsilateral spinal cord segments of the sciatic nerve and related dorsal root ganglions, the thickness of between sampled sections was 48 μm for spinal cord and 36 μm for DRG) from all rats in each experimental group were assessed by densitometry using ImageJ software. Data are expressed as a percentage of total tissue area.

### Statistical analysis

Statistical analysis was performed with SPSS Version 15. Results were presented as mean values with standard deviations. Normality of the data determined by Kolmogrov-Smirnov (K-S) normality test. Also, analysis of variance (ANOVA) followed by Tukey′s multiple comparison tests were used to assess the results. A value of *p* < 0.05 was considered significant.

## Results

### Biochemical analysis

Biochemical analysis of the MDA levels, and SOD and CAT activities for all groups is shown in Table 1. Sciatic nerve transection in the SNT group produced a significant elevation (*p* < 0.01) in lipid peroxidation level compared to SH and SH + HBO groups. The MDA levels in the SNT + pre-HBO group were significantly lower than that those in the SNT group (p < 0.01). Treatment with HBO in the SNT + post-HBO group did not produced a significant decrease in MDA levels compared to SNT group (*p* > 0.05).

**Table 1:** Effect of HBO on biochemical markers of rat spinal cord affected by sciatic nerve transection

Experimental Groups	MDA μmol/mg-protein	CAT unit/mg-protein	SOD unit/mg-protein
SH	60.67 ± 1.52	107 ± 26.87	10.33 ± 2.08
SH + HBO	60.33 ± 14.29	97 ± 8.48	9 ± 2.82
SNT	97.33 ± 1.52^**^	15 ± 2.64^***^	1.83 ± 1.25^*^
SNT + pre-HBO	65.67 ± 5.50^##^	80 ± 17.06^#^	9 ± 4.35^#^
SNT + post-HBO	90.67 ± 15.89	75 ± 19.80^#^	8.33 ± 1.52^#^

Data are represented in Mean ± SD. ^**^ *p* < 0.01 versus SH and SH + HBO; ^##^ *p* < 0. 01 versus SNT; ^***^ *p* < 0. 001 versus SH and SH + HBO; ^#^p < 0. 05 versus SNT; ^*^ *p* < 0. 05 versus SH and SH + HBO by one-way ANOVA followed by Tukey′s post-hoc tests

Sciatic nerve transection in the SNT group produced a significant (*p* < 0.001) decrease in catalase (CAT) activity compared to SH and SH + HBO groups. The CAT activities in the SNT + pre- and SNT + post-HBO groups were significantly (*p* < 0.05) higher than that in the SNT group, while the differences between SNT + Pre- and SNT + post-HBO groups were not significant (*p* > 0.05).

Sciatic nerve transection in the SNT group produced a significant (*p* < 0.05) decrease in superoxide dismutase (SOD) activity compared to SH and SH + HBO groups. The SOD activities in the SNT + Pre- and SNT + Post-HBO groups were significantly (*p* < 0.05) higher than that in the SNT group, while the differences between SNT + Pre- and SNT + Post-HBO groups were not significant (*p* > 0.05).

### Histopathologic assessment

**Fig. 1:**
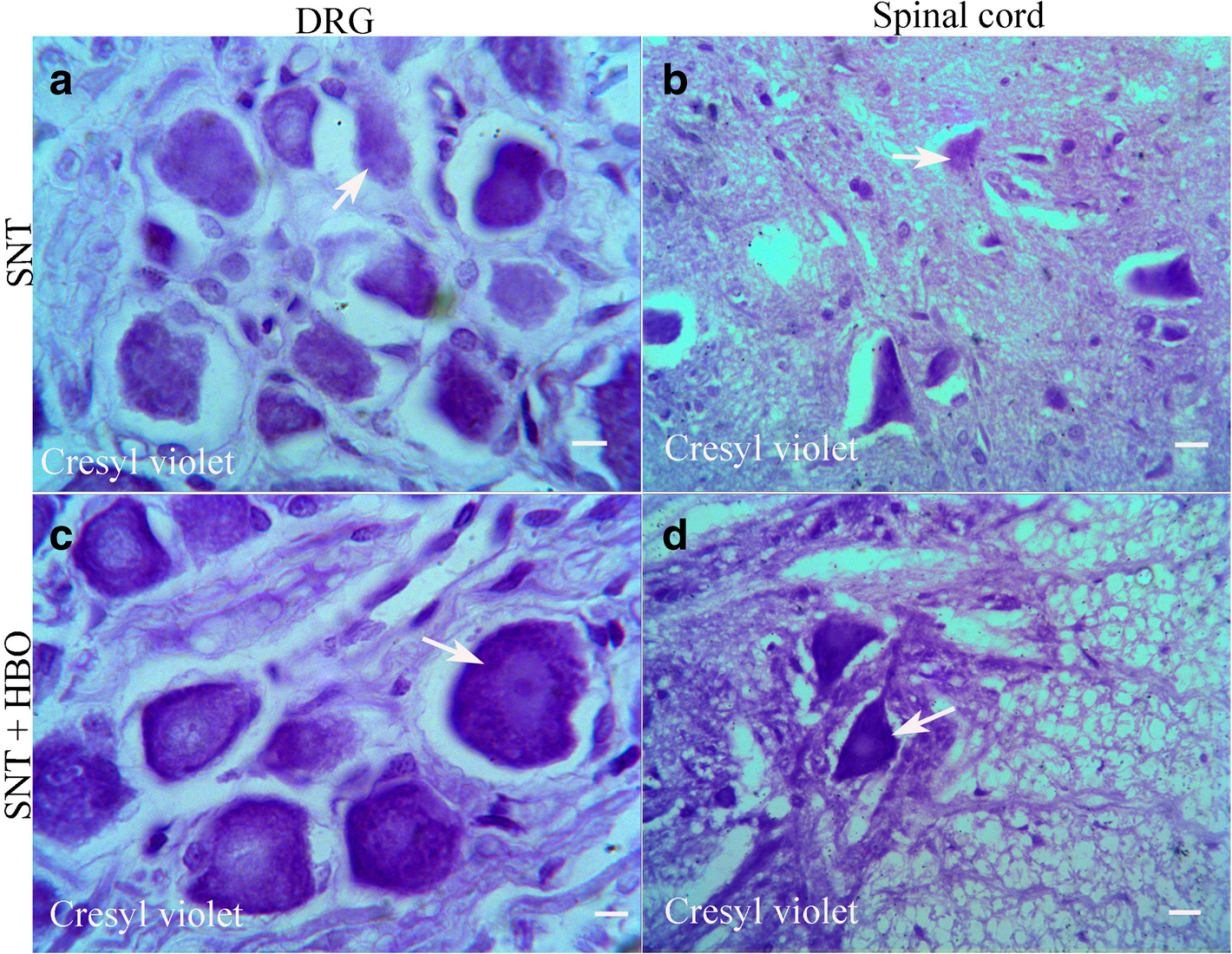
Light Photomicrographs of dorsal root ganglion (**a**) and spinal cord (**b**) horizontal section of SNT group show chromatolysis of the dorsal root ganglion neurons and anterior horn neurons of the spinal cord (arrows), while sections of dorsal root ganglion and spinal cord of SNT + HBO treated groups (**c** and **d**) show normal microscopic appearance in some of the sensory and motor neurons without chromatolysis (stained with cresyl violet; Scale bar = 100 μm)

Histological examination of the nerve-transected animals revealed cellular degeneration in sensory dorsal root ganglion neurons (Fig. 1a) and in spinal cord motoneurones (Fig. 1b). The changes include dissolution of Nissl bodies and displacement of the nucleus to the periphery, chromatolysis. Treatment with HBO in the SNT + Pre- and SNT + Post-HBO groups reduced the changes; so that normal microscopic appearance in some of neural cells was detected in dorsal root ganglion (Fig. 1c) and spinal cord (Fig. 1d). No detectable injury was shown in SH and SH + HBO groups.

### Immunohistochemical assessment

**Fig. 2:**
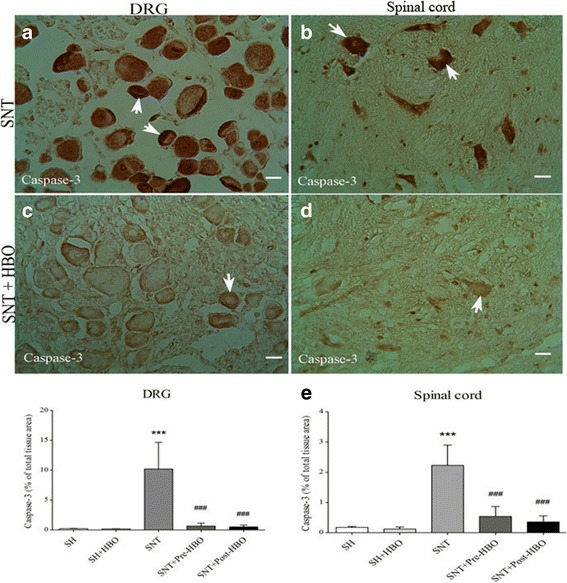
Light Photomicrographs show immunohistochemical staining of caspase-3 in dorsal root ganglion neurons and anterior horn neurons of the spinal cord in SNT (**a** and **b**) and SNT + HBO treated (**c** and **d**) groups, respectively. The positive staining of caspase-3 is presented by a brown color of cytoplasm (arrows) (Scale bar = 100 μm). The intensity of the immunohistochemical staining in sensory and motor neurons were decreased after HBO treatment. Densitometry analysis of immunohistochemical photomicrographs for caspase-3 was assessed (**e**). Data are expressed as a percentage of total tissue area. ^***^ *P* < 0.001 versus SH and SH + HBO groups; ^###^P < 0.001 versus SNT group by one-way ANOVA followed by Tukey′s post-hoc tests

Nerve transection in the SNT group increased the expression of caspase-3 in dorsal root ganglion (Fig. 2a) and spinal cord (Fig. 2b) compared to SH and SH + HBO groups. HBO treatment in the SNT + Pre- and SNT + Post-HBO groups reduced the degree of positive staining for caspase-3 in dorsal root ganglion (Fig. 2c) and spinal cord (Fig. 2d) compared to SNT group. Quantitative analysis of caspase-3 positive staining in the experimental groups is shown in Fig. 2e.

**Fig. 3:**
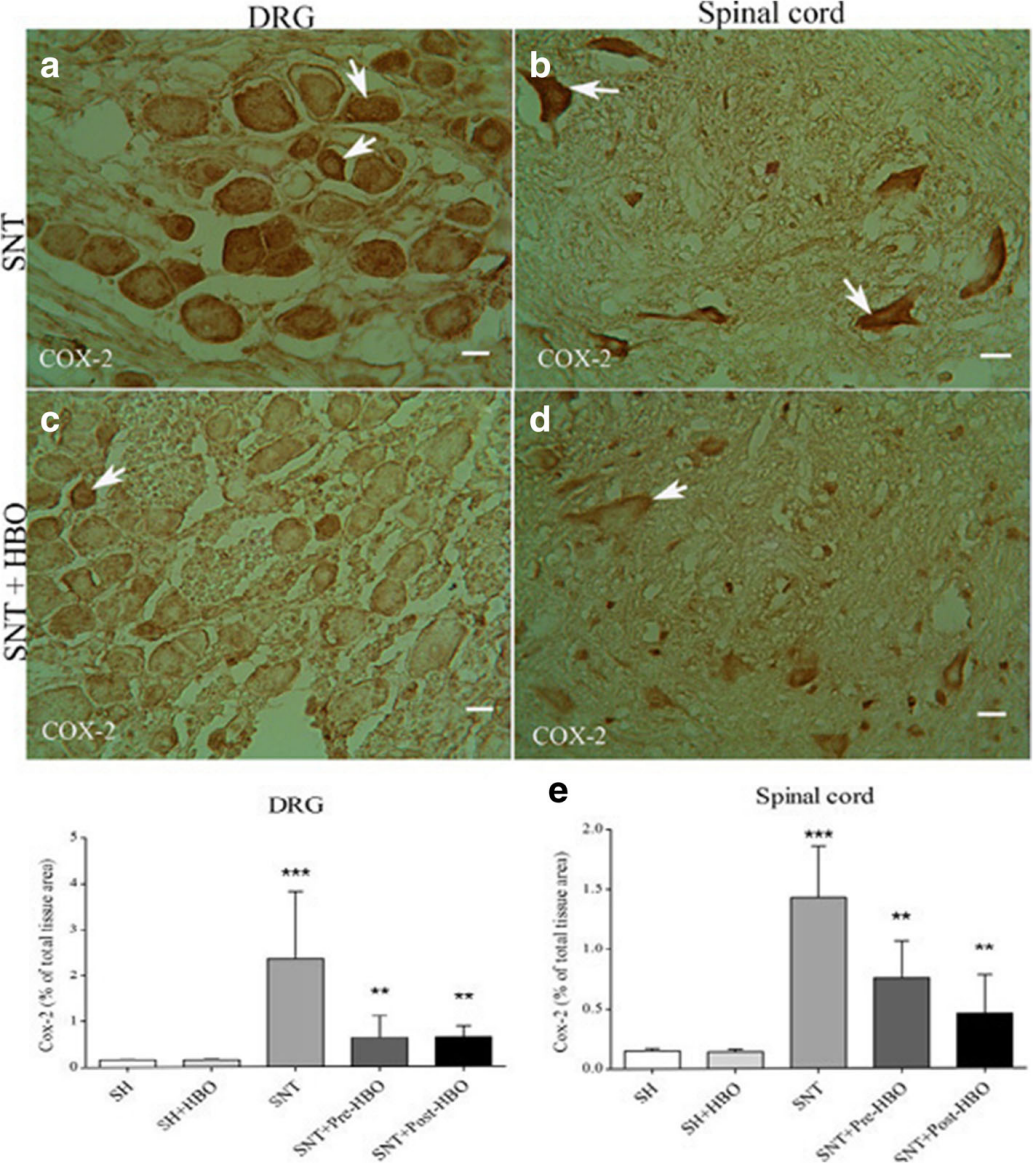
Light Photomicrographs show immunohistochemical staining of COX-2 in dorsal root ganglion neurons and anterior horn neurons of the spinal cord in SNT (**a** and **b**) and SNT + HBO treated (**c** and **d**) groups, respectively The positive staining of COX-2 is presented by a brown color of cytoplasm (arrows) (Scale bar = 100 μm). The intensity of the immunohistochemical staining in sensory and motor neurons were decreased after HBO treatment. Densitometry analysis of immunohistochemical photomicrographs for COX-2 was assessed (**e**). Data are expressed as a percentage of total tissue area. ^***^ *P* < 0.001 versus SH and SH + HBO groups; ^**^ *P* < 0.01 versus SNT group by one-way ANOVA followed by Tukey′s post-hoc tests

Nerve transection in the SNT group increased the expression of COX-2 in dorsal root ganglion (Fig. 3a) and spinal cord (Fig. 3b) compared to SH and SH + HBO groups. HBO treatment in the SNT + Pre- and SNT + Post-HBO groups reduced the degree of positive staining for COX-2 in dorsal root ganglion (Fig. 3c) and spinal cord (Fig. 3d) compared to SNT group. Quantitative analysis of COX-2 positive staining in the experimental groups is shown in Fig. 3e.

**Fig. 4:**
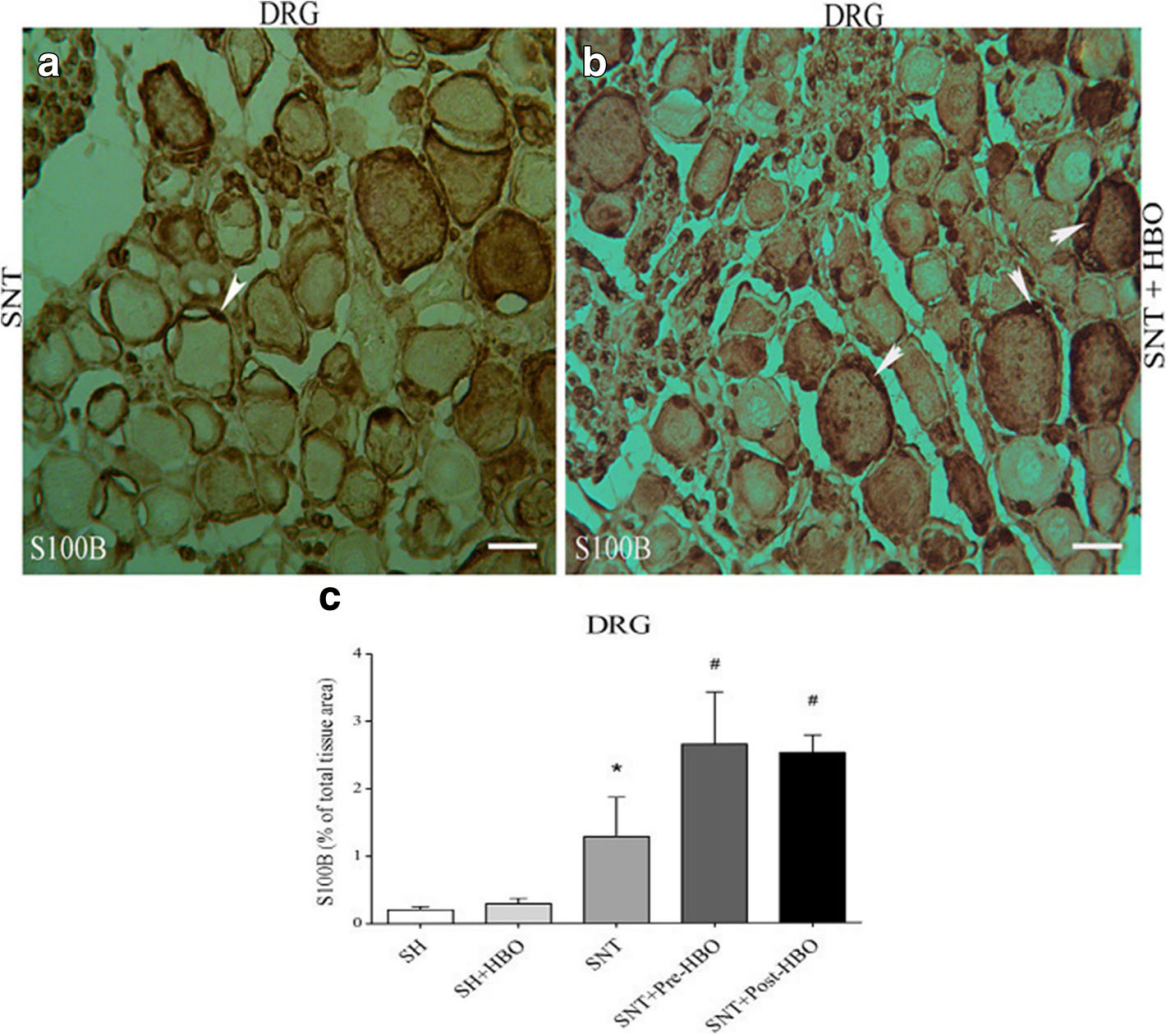
Light Photomicrographs show immunohistochemical staining of S100ß in dorsal root ganglion neurons of SNT (**a**) and SNT + HBO treated (**b**) groups, respectively. The positive staining of S100ß is presented by a brown color of cytoplasm (arrows) (Scale bar = 100 μm). The intensity of the immunohistochemical staining in sensory neurons were decreased after HBO treatment. Densitometry analysis of immunohistochemical photomicrographs for S100ß was assessed (**c**). Data are expressed as a percentage of total tissue area. ^*^ *P* < 0.05 versus SH and SH + HBO groups; ^#^P < 0.05 versus SNT group by one-way ANOVA followed by Tuke’s post-hoc tests

Nerve transection in the SNT group increased the expression of S100ß, a marker of Satellite and Schwann cells, in dorsal root ganglion (Fig. 4a) compared to SH and SH + HBO groups. HBO treatment in the SNT + Pre- and SNT + Post-HBO groups increased the degree of positive staining for S100ß in dorsal root ganglion (Fig. 4b) compared to SNT group. Quantitative analysis of S100ß positive staining in the experimental groups is shown in Fig. 4c.

### TUNEL assessment

**Fig. 5:**
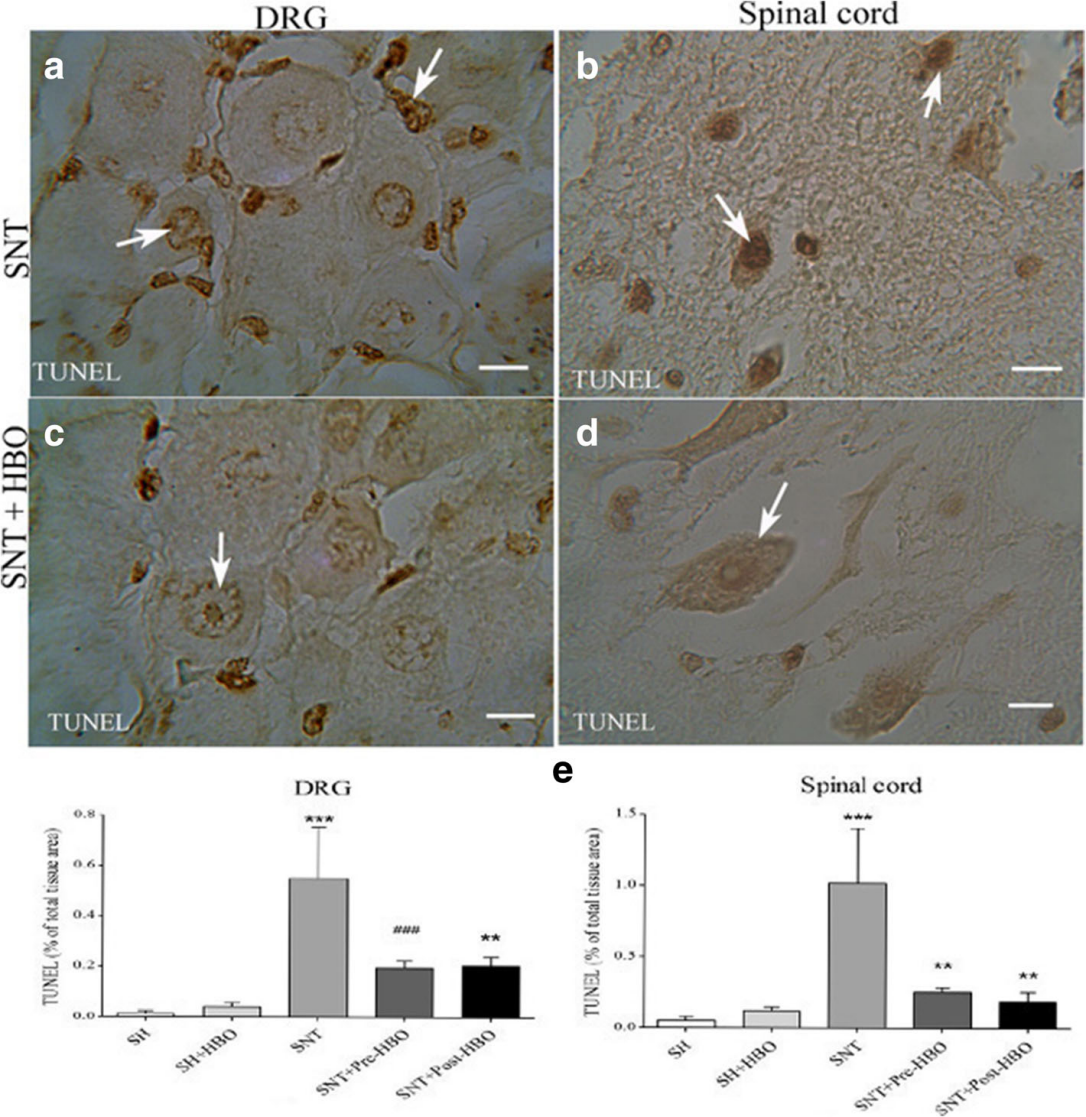
Light Photomicrographs show TUNEL-positive cells in dorsal root ganglion neurons and anterior horn neurons of the spinal cord in SNT (**a** and **b**) and SNT + HBO treated (**c** and **d**) groups, respectively. The positive staining of TUNEL is presented by a brown color of nucleus (arrows) (Scale bar = 100 μm). The intensity of the TUNEL staining in sensory and motor neurons were decreased after HBO treatment. Densitometry analysis of photomicrographs for TUNEL reaction was assessed (**e**). Data are expressed as a percentage of total tissue area. ^***^P < 0.001 versus SH and SH + HBO groups; ^**^P < 0.01 versus SNT group; ^###^P < 0.001 versus SNT group by one-way ANOVA followed by Tukey’s post-hoc tests

Almost no TUNEL-positive cells could be detected in SH and SH + HBO groups, whereas many cells were intensely stained in the dorsal root ganglion (Fig. 5a) and spinal cord (Fig. 5b) obtained from SNT group. In contrast, a small number of TUNEL-positive cells were detected in dorsal root ganglion (Fig. 5c) and spinal cord (Fig. 5d) obtained from HBO pre- and post-treated rats. Quantitative analysis of TUNEL- positive staining in the experimental groups is shown in Fig. 5e.

## Discussion

The current study indicated that hyperbaric oxygen therapy promotes neuron survival through attenuating apoptosis, inflammation, and lipid peroxidation, and also through improving antioxidant status after sciatic nerve transection.

Our immunohistochemical results showed that sciatic nerve transection considerably increased the expression of caspase-3 in sensory dorsal root ganglion neurons and in spinal cord motoneurones, which plays a critical role in apoptosis. On the contrary, our results showed that these up regulations significantly attenuated after HBO treatment, while the differences between pre- and post-treatment were not significant. To correlate neuronal cell apoptosis, we carried out TUNEL staining method. Retrograde neuronal apoptosis, which occurs in sensory dorsal root ganglion neurons and in spinal cord motoneurones, is one contributing factor of poor sensory recovery and reduced motor function after peripheral nerve transection [3, 36]. On the other hand, the survival of the neurons following injury is of great importance for the outcome of the axonal regeneration and target organ reinnervation. In this regard, it is well known that neuronal cell apoptosis after peripheral nerve transection is mediated through expression of apoptosis-related genes namely Bax, Bcl2, and caspase-3 [37, 38]. Several studies have shown that HBO prevents apoptosis in experimental neurological disorder models. In this regard, it was found that HBO therapy prevented apoptosis through opening of the mitochondrial ATP-sensitive potassium channels [39], decreasing of caspase-3 [40], and decreasing of phosphorelated-p38 mitogen-activated protein kinas [41] in ischemic brain. Also, it was documented that HBO therapy prevented apoptosis through decreasing of hypoxia-inducible factor-1α (HIF-1α) [42], adaptor molecule apoptosis-associated speck-like protein (ASC), and caspase-3 [43, 44] after spinal cord injury. Recently, it was found that hyperbaric oxygen therapy following chronic sciatic nerve constriction injury produced antinociceptive effects through different mechanisms such as downregulation of caspase-3 and inhibition of apoptosis [32].

Our immunohistochemical results showed that sciatic nerve transection considerably increased the expression of COX-2 in dorsal root ganglion and spinal cord. On the contrary, our results showed that these up regulations significantly attenuated after HBO treatment compared to non-treated rats, while the differences between pre- and post-treatment were not significant. Following peripheral nerve injury, various inflammatory mediators were upregulated in spinal cord and dorsal root ganglion [45, 46], which have been implicated in the axonal degerative process after injury. One of the inflammatory mediators that play an important role during these processes is cyclooxygenase-2 (COX-2) [47]. Several lines of evidence have shown that HBO treatment exerts neuroprotective effects via mechanisms such as inhibition of inflammation. In this regard, it was found previously that the antinociceptive effect of HBO treatment is associated somewhat with anti-inflammatory properties in a rat model of neuropathic pain [48]. HBO treatment decreased NF-kB, IL-1ß, and TNF-α levels after spinal cord injury [12]. Another study reported that HBO reduced COX-2 level in ischemic cerebral tissue [49].

In the present study, HBO treatment decreased malondialdehyde (MDA) levels as an index of lipid peroxidation in spinal cord of pre-treatment group compared to non-treated rats, while the difference in post-treatment group was not significant. Therefore, HBO pre-treatment was more effective than HBO post-treatment against lipid peroxidation. Also, HBO treatment increased catalase and superoxide dismutase activities as endogenous antioxidants in spinal cord of pre- and post-treatment groups compared to non-treated rats, while the differences between pre- and post-treatment were not significant. Free radical-induced lipid peroxidation is the primary pathway of peripheral nerve injury [50], which is elevated in the spinal cord after sciatic nerve transection [51]. Evidences also affirm that pheripheral nerve transection leads to pro-oxidative status in the spinal cord due to a decrease in antioxidant enzyme activities [52]. Studies revealed the alternation of enzymatic antioxidant activity after HBO exposure. Li et al. [21] reported that HBO induced tolerance against brain ischemia-reperfusion injury by upregulation of antioxidant enzyme activity of catalase and superoxide dismutase. Also, it was found that HBO enhanced superoxide dismutase activity and reduced tissue damage after hypoxia-ischemia brain damage in neonatal rats [53].

Our immunohistochemical results showed that sciatic nerve transection increased the expression of S100ß in the dorsal root ganglion. Paradoxically, we found that HBO applied before or after SNT further increased S100ß expression significantly compared to SNT alone. Therefore, since it is well known that S100ß acts as an inhibitor of apoptosis and a stimulator of cell proliferation in neurodegenerative process [54], the increase in S100ß induced by SNT alone could be viewed as a compensatory mechanism of the body to deal with apoptosis after SNT (the present study) and other neural injuries [55, 56]. In this regard, previous studies have shown that S100ß expression upregulated in dorsal root ganglion after sciatic nerve transection [55]; so that treatment with S100ß decreased neural death after sciatic nerve transection [56]. Zhang et al. [57] found that HBO therapy after traumatic brain injury reduced neuronal loss and increased expression of S100 astrocyte marker in brain tissue.

## Conclusions

The biochemical, histopathological, and immunohistochemical evidences demonstrated that pre- and post-HBO therapy had neuroprotective effects against sciatic nerve transection-induced degeneration. On the other hand, the results propose that HBO treatment is effective in protection of sensory and motor neurons against retrograde apoptosis and enhance neuronal survival time after peripheral nerve transection.

## Abbreviations

CAT: Catalse
COX-2: Cyclooxigenase-2
HBO: Hyperbaric oxygen
MDA: Malodialdehyde
S100ß: S100beta
SH: Sham-operated
SNT: Sciatic nerve transaction
SOD: Superoxide dismutase
TUNEL: Terminal deoxynucleotidyl transferase dUTP nick end labeling
